# Integrative Korean medicine for recurrent lumbar disc herniation after coronavirus disease vaccination: A case report and literature review

**DOI:** 10.1097/MD.0000000000041079

**Published:** 2025-01-03

**Authors:** Ah-Ra Koh, Hyun-Woo Kim, Young-Jin Lee, Hye-Jeong Jo, Go-Eun Chae, Dong-Woo Kim, In-Hyuk Ha, Doori Kim

**Affiliations:** a Department of Korean Medicine Rehabilitation, Ulsan Jaseng Hospital of Korean Medicine, Ulsan, Republic of Korea; b Department of Acupuncture & Moxibustion Medicine, Ulsan Jaseng Hospital of Korean Medicine, Ulsan, Republic of Korea; c Department of Internal Korean Medicine, Ulsan Jaseng Hospital of Korean Medicine, Ulsan, Republic of Korea; d Jaseng Spine and Joint Research Institute, Jaseng Medical Foundation, Seoul, Republic of Korea; e Centor for Clinical Research, Jaseng Hospital of Korean Medicine, Seoul, Republic of Korea.

**Keywords:** case report, coronavirus disease vaccination, COVID-19, integrative Korean medicine, recurrent lumbar disc herniation

## Abstract

**Rationale:**

Many side effects have been associated with the coronavirus disease (COVID-19) vaccine. While most adverse events (AEs) are mild, serious adverse events are occasionally observed in the neurological and musculoskeletal systems. Nevertheless, articles reporting such serious adverse events after COVID-19 vaccination are limited, and only few case reports with detailed descriptions are available in the literature.

**Patient concerns:**

Herein, we report the case of a 41-year-old male office worker who developed symptoms of recurrent disc herniation 2 days after COVID-19 vaccination; the patient had no other factors that may have caused the recurrence of disc herniation, such as excessively vigorous activities, following vaccination.

**Diagnoses:**

Consequently, the patient was suspected of having cauda equina syndrome owing to recurrent lumbar disc herniation, and he underwent surgery.

**Interventions:**

The patient underwent integrative Korean medicine treatment, including acupuncture, pharmacopuncture, and Chuna manual therapy, for 8 months postoperatively.

**Outcomes:**

After treatment, the patient’s postoperative complications improved; the Numerical Rating Scale score changed from 5 to 1, and the Oswestry Disability Index score changed from 30 to 3. A literature review showed various cases of adverse events related to musculoskeletal inflammation or immune-mediated pathogenesis.

**Lessons:**

This paper confirmed the possibility that COVID vaccination is related to lumbar disc herniation recurrence and the possibility of integrative Korean medicine as an effective treatment option after lumbar disc herniation surgery.

## 1. Introduction

Lumbar disc herniation (LDH) occurs when the lumbar intervertebral disc degenerates or an external force causes fissures or tears of the central, inner, or outer collagen fibers of the annulus fibrosus; this causes herniation of part or the entire nucleus pulposus, which compresses the dura or nerve root and causes neurological symptoms.^[1]^ Recurrent lumbar intervertebral disc herniation (recurrent LDH [rDH]) is not uncommon, and its management costs approximately $26,593 per patient.^[2]^ Risk factors for rDH include obesity, smoking, male sex, diabetes, hypertension, and occupational heavy lifting.^[3]^ The incidence of postoperative rDH is reportedly 2% to 25%^[4]^; the recurrence rate within 1 year for those with occupational exposure is 60%.^[5]^

Conservative treatment is the first-line approach for managing LDH; however, if symptoms such as urinary and/or fecal incontinence, progressive weakness, and radicular pain persist even after 4 to 6 weeks of conservative therapy, surgery is recommended.^[6]^ In the United States (US), lumbar discectomy is the most common treatment modality for LDH with radicular pain in clinical practice.^[7]^ Surgery is indicated for treating LDH in cases where acute bladder and bowel impairment is present or if the sciatica is incapacitating and persists for >6 to 12 weeks.^[8]^ Surgery is considered the standard treatment for recurrent LDH.^[4]^

However, surgical treatment outcomes are not always favorable.^[9]^ Failed back surgery syndrome (FBSS) refers to suboptimal outcomes following spinal surgery.^[10]^ In a long-term follow-up of 10 to 22 years in patients who underwent standard lumbar discectomy for LDH, 74.6% of patients experienced recurring low back pain (LBP) and reoperation was required in 12% of cases.^[11]^ Conservative treatment options for FBSS include spinal cord stimulation, medication, epidural steroid injections, physical therapy, and cognitive-behavioral therapy.^[12]^

In Korea, integrative Korean medicine (IKM) is an alternative treatment for FBSS. According to a study of 707 patients with FBSS who visited a Korean medicine hospital, 47% chose IKM as their primary treatment for postoperative symptoms.^[13]^ In a 1-year observational study of 120 patients with FBSS who received IKM treatment, 79.2% reported an improvement in their quality of life, confirming the long-term effects of IKM treatment.^[14]^ Furthermore, the clinical efficacy of IKM treatment was demonstrated in patients with acute FBSS for <1 month.^[15]^ However, high-quality evidence on the effects of IKM treatment on FBSS is lacking, and the efficacy of KM manual therapy in treating FBSS remains unconfirmed.^[16]^

Coronavirus disease (COVID-19) is an infectious disease caused by severe acute respiratory syndrome coronavirus 2. The first outbreak of COVID-19 was reported in Wuhan, China, in 2019,^[17]^ and the pandemic has been regarded as the most disruptive event in recent world history, resulting in >500 million confirmed cases and >6.3 million deaths in approximately 3 years.^[18]^ COVID-19 vaccines were developed far more rapidly than traditional vaccines, with a reported timeline of 12 to 16 months in development and rollout under Emergency Use Authorization in many major countries, compared with the usual 10 to 15 years of development for traditional vaccines.^[19]^

Following this expedited process of development and rollout, various sequelae ranging from mild to serious adverse events (SAEs) have been reported after COVID-19 vaccination. The reported common adverse events (AEs) include pain at the injection site, redness, itchiness, nausea, and joint pain^[20]^; however, in rare cases, SAEs with neurological and musculoskeletal manifestations are also observed. Among the AEs reported to the US Vaccine Adverse Event Reporting System (VAERS) between 2019 and September 23, 2023, 450 cases of LDH were reported.^[21]^ According to the Pfizer (Pfizer Inc., New York City, NY)/BioNTech (BioNTech SE, Mainz, Germany) Vaccine Analysis by the UK Medicines and Healthcare Products Regulatory Agency, which covered reports of adverse drug reactions between December 9, 2020 and April 20, 2022, there was one case each of acquired syringomyelia, cauda equina syndrome (CES), myelopathy, and brachial radiculitis in the category of spinal cord and nerve root disorders.^[22]^ Moreover, among the AEs reported to the VAERS in the US since the COVID-19 outbreak in 2019, there were 25 cases of CES,^[21]^ and some studies reported LDH as a postvaccination AE.^[23,24]^ However, limited research exists on SAEs following COVID-19 vaccination. To the best of our knowledge, few case reports with detailed descriptions have been published.

Herein, in this study, we aim to report the possible AEs of COVID-19 by introducing a case in which LDH recurred and worsened after COVID-19 vaccination and reviewing the related literature. In addition, we would like to introduce the successful treatment and management of symptoms after LDH surgery using Korean medicine.

## 2. Case presentation

### 2.1. Chief complaints

A 41-year-old male office worker visited our hospital on December 22, 2022, with the chief complaints of LBP, lower extremity (LE) pain, and numbness. The patient was admitted and received inpatient treatment from December 23, 2021 to January 7, 2022.

### 2.2. History of present illness

On August 24, 2021, the patient was confirmed to be COVID-19-positive and underwent negative pressure therapy. The infectious disease was completely resolved, and he was discharged on September 1. On December 17 of the same year, he received the first dose of the BNT162 vaccine from Pfizer/BioNTech. Two days later, he experienced the onset of symptoms.

### 2.3. History of past illness

**Figure 1.**
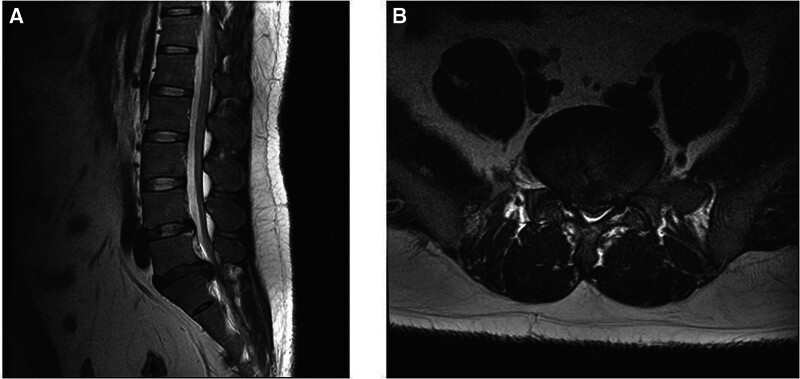
(August 05, 2011) L-spine magnetic resonance imaging. (A) T2 sagittal view; (B) T2 axial view.

The patient had sudden onset of pain in the right buttock and radicular pain in the right LE during daily activities on July 24, 2011. Lumbar magnetic resonance imaging (MRI) scans obtained on August 5, 2011 revealed a large L5/S1 disc herniation (Fig. 1) and surgery was recommended. However, he refused to undergo surgery, was admitted to our hospital on August 6, 2011, with a chief complaint of numbness from the right buttock to the second, third, fourth, and fifth toes, and received inpatient care from August 8 to August 21. After discharge, the patient complained of discomfort and continued treatment with epidural selective nerve root steroid injection and outpatient IKM treatment. After the last treatment on February 25, 2012, the symptoms disappeared, and the treatment was discontinued.

### 2.4. Physical examination upon admission

During his visit on December 22, 2021, the patient complained of pain in the lumbosacral region (from the lower lumbar region to the sacral region), radicular pain, and hypesthesia (numbness) along the back of both LEs to the tarsal area. The tingling sensation down the back of the left LE and hypesthesia were particularly severe.

On December 24, 2021, day 2 of his hospital stay, the patient developed constipation and was prescribed herbal medicines for treatment. On December 26, the patient reported aggravation of his abdominal discomfort, sensation of incomplete bladder emptying, and voiding difficulty. After consultation at the Department of Internal Medicine, he was prescribed a 7-day medication course for constipation. After taking the medication, he was able to pass the stools, but still experienced frequent urination and a sensation of incomplete bladder emptying. Meanwhile, the patient showed temporary improvement in pain and numbness, with a Numerical Rating Scale (NRS) score of 2 to 3 for the LE radicular pain.

After consultation with the Department of Urology on December 27 and 31, 2021, the patient underwent urinary catheterization and was prescribed medication for voiding dysfunction. There was slight improvement in the incomplete bladder emptying sensation; however, the patient still had reduced sensation around the perineum. CES was suspected, and surgery was recommended; however, the patient was hesitant about undergoing surgery. Therefore, we decided to monitor symptom progression for the time being. On January 6, 2022, after removing the urinary catheter, the patient succeeded in self-voiding once and urinated 7 to 8 times during the night. Urination frequency slightly improved compared to that before catheterization, but the volume remained small. Limitations in mobility persisted due to high pain levels during walking.

### 2.5. Imaging examinations

**Figure 2.**
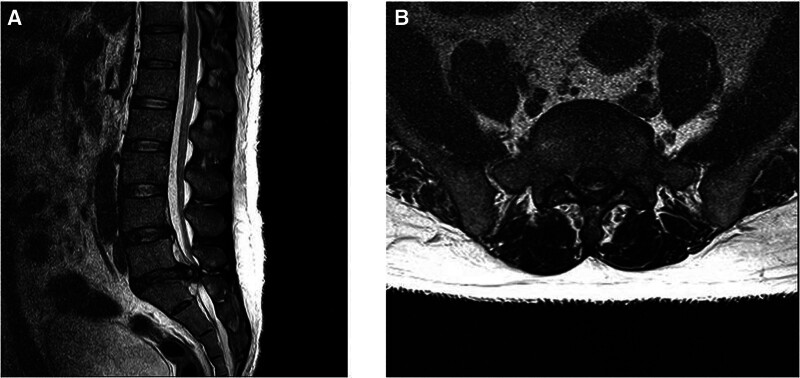
(January 07, 2022) L-spine magnetic resonance imaging: preoperatively. (A) T2 sagittal view; (B) T2 axial view.

On January 7, 2022, the patient reported persistent pain in the LE, starting from the left buttock, and a feeling of drop in his foot due to Bi Jeung (numbness or pain). Owing to persistent LE weakness and no pain reduction, surgery was deemed necessary, and the patient was transferred to a hospital for surgical treatment. A lumbar spine MRI scan performed on January 7, following discharge, revealed focal central extrusion at L5–S1 with inferior migration and moderate-to-severe central canal stenosis (Fig. 2).

**Figure 3.**
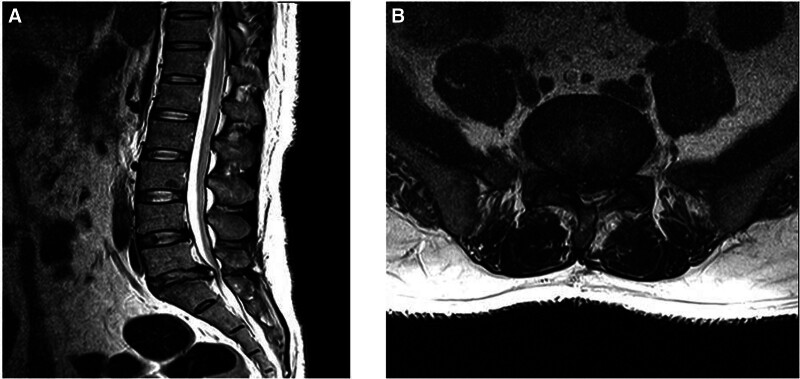
(January 14, 2022) L-spine magnetic resonance imaging: postoperatively. (A) T2 sagittal view; (B) T2 axial view.

On January 11, microendoscopic discectomy and partial laminectomy were performed at the L5–S1 disc level. Lumbar spine MRI scan findings obtained postoperatively on January 14 showed that most of the herniated intervertebral disc had been removed; however, there was a possibility of a postoperative change/remnant disc (Fig. 3). Postoperatively, the patient’s previous pain improved, but hypesthesia along the back of the left thigh and tingling in the sole of his foot persisted, with no complete recovery of gait. The patient was discharged from the hospital on February 10 with a slight drop in his foot owing to reduced muscle strength in his left foot, particularly when using stairs or ramps.

**Figure 4.**
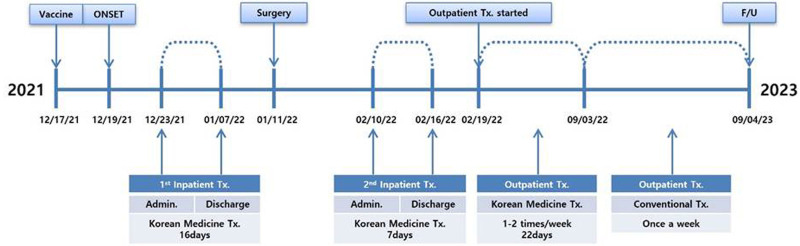
Patient timeline. Admin. = administration, F/U = follow-up, Tx = treatment.

The patient revisited the hospital on February 10, 2022, complaining of LBP, LE pain, and numbness. He had increasing Bi Jeung and hypesthesia in the posterior region of both thighs when sitting, along with worsening pain in the left calf during walking. He received inpatient treatment until February 16, 2022. Then, he was discharged, and outpatient treatment was continued. The treatment timeline is summarized in Figure 4.

### 2.6. Final diagnosis

The final diagnosis was CES.

### 2.7. Treatment

The patient underwent microendoscopic discectomy and partial laminectomy for the surgical treatment of CES. He also underwent IKM treatment, including acupuncture, herbal medicine, pharmacopuncture, moxibustion, cupping, and Chuna manual therapy (CMT), for the management of postoperative symptoms in inpatient and outpatient care settings. The patient continued outpatient visits with manual therapy alone. Tables 1–4 present details of the individual modes of treatment and frequency of treatment sessions for the first and second admissions, surgical treatment period, and outpatient treatment, respectively. Information on the herbal treatments is presented in Tables S1–S3, Supplemental Digital Content, http://links.lww.com/MD/O243.

**Table 1:** Details of intervention during the first inpatient treatment (December 23, 2021–January 07, 2022).

Treatment type	Treatment details and frequency
Herbal medicine	
Cheongpajeon December 23, 2021 to January 07, 2022	30 minutes after each meal, 3 times a day (TID), for 16 days
Chungsinbaro-Hwan December 23, 2021 to January 07, 2022	30 minutes after each meal, TID, for 16 days
Gwanjul-Go December 23, 2021 to January 07, 2022	30 minutes after each meal, TID, for 16 days
Madae-Hwan December 24, 2021 to December 25, 2021	30 minutes after each meal, twice a day (BID), for 2 days
Pharmacopuncture	
Shinbaro (Jaseng Korean Medical Hospital, Namyangju, Republic of Korea) December 23, 2021 to January 07, 2022	BID, disposable syringe (Kovax-Syringe 2 mL, 26 G × 1 1/2), for 16 days
Chuna manual therapy	
December 23, 2021 to January 07, 2022	Side lying position, lumbar distraction technique, prone position, sacral dysfunction correction technique, prone position, iliac dysfunction correction technique, once a day, for 16 days

**Table 2:** Details of interventions performed before, during, and after surgery (January 07, 2022–February 10, 2022).

Treatment type	Treatment details and frequency
Injection	
January 01, 2022	Fentanyl Citrate Inj. 500 mcg/10 mL, Dexamethasone Inj. 5 mg, Lidocaine hydrochloride (HCL) Hydrate Inj. 5 mL, Cefazedone Sodium Inj. 1g-Kyongbo, Ramset Prefilled Inj. 0.3 mg/2 mL, Remiva Inj. 2 mg, Rocunium Inj. 5 mL, Anepol Inj. 5 0 mg/5 mL, Pigmin Inj. 5 mg/mL, Tabinul Inj. 200 mcg/mL, Sodium Bicarbonate Inj. 8.4% 20 mEg/20 mL, Marobiven-A Inj., Botropase Inj. 2 KU, Samjin Taurolin Inj. 2% 250 mL, Ciprofloxacin Inj. 100 mL, Amoburofen Inj. 800 mg/8 mL, H-2 Inj., Tramadol HCl Inj. 50 mg/mL, Rosiden Inj. 20 mg/1 mL/1A, Triam Inj. 40 mg/mL, Huons Lidocaine HCl Inj. 2% 2 mL
Intervention/surgery	
January 07, 2022 January 08, 2022 January 11, 2022	Urinary catheterization Indwelling urinary catheterization Lumbar discectomy (open technique)
Physical treatment	
January 24, 2022 to February 09, 2022	Interferential current therapy, superficial/deep heat therapy, once a day, for 12 days

**Table 3:** Details of intervention during the second inpatient treatment (February 10, 2022–February 16, 2022).

Treatment type	Treatment details and frequency
Herbal medicine	
Cheongpajeon February 10, 2022 to February 16, 2022	30 minutes after each meal, twice a day (BID), for 7 days
Pharmacopuncture	
Shinbaro (Jaseng Korean Medical Hospital, Namyangju, Republic of Korea) February 10, 2022 to February 16, 2022	BID, disposable syringe (Kovax-Syringe 2 mL, 26 G × 1 1/2), for 7 days
Chuna manual therapy (CMT)	
February 10, 2022 to February 16, 2022	Side lying position lumbar distraction technique, prone position sacral dysfunction correction technique, prone position iliac dysfunction correction technique Once a day, for 7 days

**Table 4:** Details of intervention during outpatient treatment (February 19, 2022–September 03, 2022).

Treatment type	Treatment details and frequency
Herbal medicine	
Cheongpajeon March 12, 2022	30 minutes after each meal, twice a day, for 15 days
Pharmacopuncture	
Shinbaro (Jaseng Korean Medical Hospital, Namyangju, Republic of Korea) February 19, 2022 to September 03, 2022	Once a day (QD), disposable syringe (Kovax-Syringe 2 mL, 26 G × 1 1/2), for 22 days
Chuna manual therapy (CMT)	
February 19, 2022 to May 06, 2022	Side lying position lumbar distraction technique, prone position sacral dysfunction correction technique, prone position iliac dysfunction correction technique QD, for 9 days

For the herbal treatments, Cheongpajeon, Chungsinbaro-hwan, and Gwanjul-go were used, all effective in treating musculoskeletal diseases.

Pharmacopuncture was used to release adhesions between the L5–S1 nerve root and surrounding tissues. Acupoints around both lower lumbar regions and sacroiliac joint and those around the tender points of both buttocks were mainly used; Shinsu (BL23), Gihaesu (BL24), Taejangsu (BL25), Shangliao (BL31), and Hwando (GB30) were used for intensive treatment on the left side. For general acupuncture, electroacupuncture was performed at a 3-Hz frequency for 15 minutes using the same acupoints as in pharmacopuncture.

CMT was used to correct and adjust any malalignment of the lumbar spine, specifically to promote and ease the movement of the joints between the lower back and pelvic region and relieve muscle tension. Experienced Korean medicine doctors administered the CMT; manipulation techniques were used depending on the patient’s symptoms.

During the inpatient and outpatient treatment periods, conventional treatment, including physical therapy, extracorporeal shock wave therapy, and manual therapy, was administered.

## 3. Outcome and follow-up

**Figure 5.**
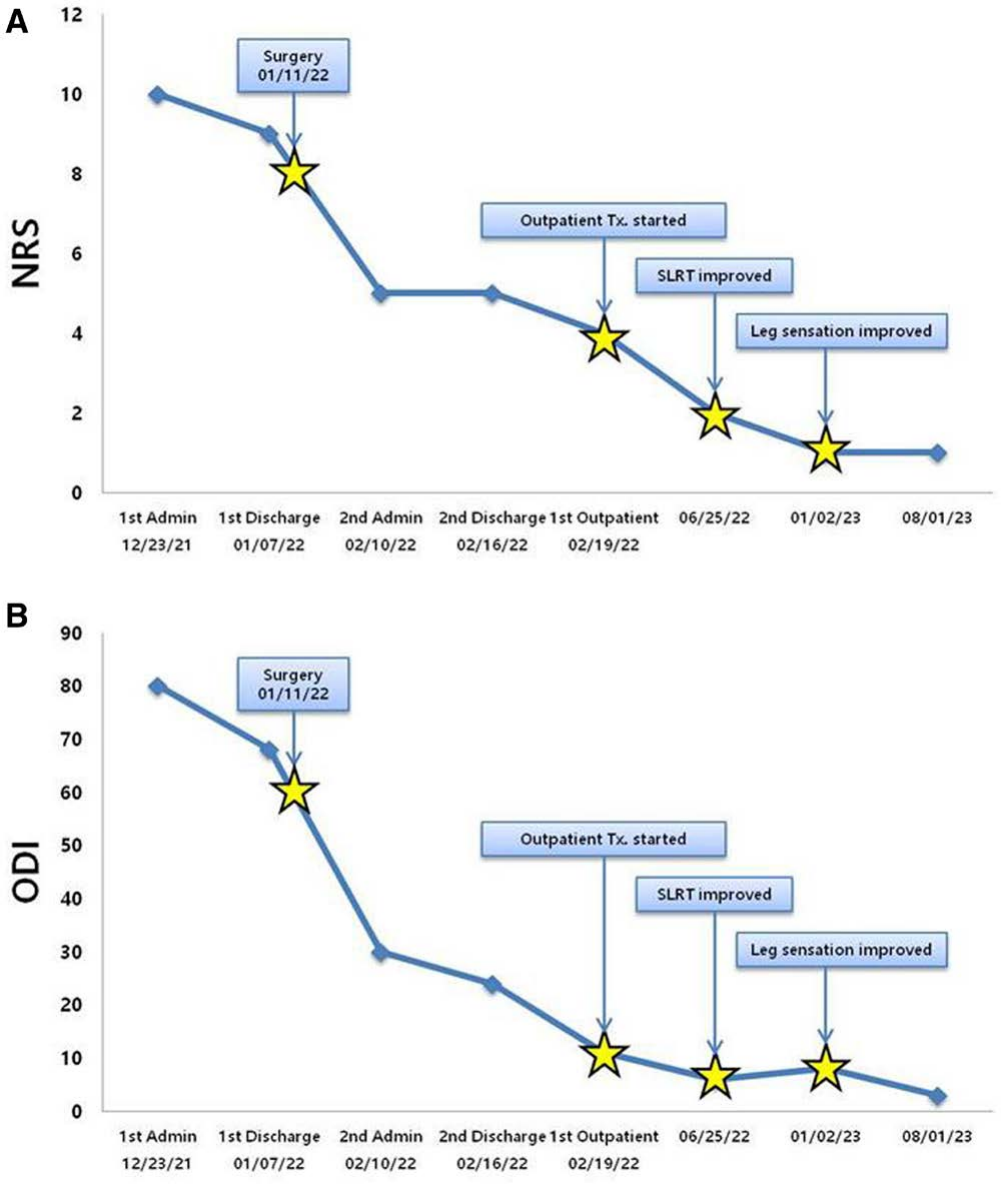
Changes in outcomes for radiculopathy. (A) Numerical Rating Scale (NRS); (B) Oswestry Disability Index (ODI). Admin = administration, SLRT = Straight Leg Raise Test, Tx = treatment.

The major changes in patient outcomes are summarized in Table 5 and illustrated in Figure 5.

**Table 5:** Changes in major outcomes and improvements in sensory function and muscle strength.

	NRS	ODI	EQ-5D	Hypoesthesia and tingling	SLRT
December 23, 2021 First admission	10	80	0.316	Hypoesthesia in the posterior region of both LEs (Lt > Rt, 40% of normal sensation)	20/20
January 07, 2022 First discharge	9	68	0.316	Hypoesthesia in the posterior region of both LEs (Lt > Rt, 40% of normal sensation)	20/20
February 10, 2022 Second admission after surgery	5	30	0.765	Bi Jeung in the posterior region of both LEs (Lt > Rt) Pain/Bi Jeung in the posterior region of the left calf muscle (gastrocnemius muscle)	40/40
February 16, 2022 Second discharge	5	24	0.795	Bi Jeung in the posterior region of both LEs (Lt > Rt) Pain/Bi Jeung in the posterior region of the left calf muscle	40/40
February 19, 2022 First outpatient treatment after discharge	4	11		Hypoesthesia when touching the posterior region of both thighs (Rt > Lt) Persistent tingling sensation in the feet (sole) on both sides (Rt > Lt)	AROM 20/20 PROM 30/30
June 25, 2022	2	6		Reduction in the affected area of hypesthesia when touching the posterior region of both thighs Disappearance of persistent tingling sensation in the feet on both sides	AROM 50/50 PROM 60/60
January 02, 2023	1	8		Improvement of hypesthesia in the left LE region	
August 01, 2023	1	3		Improvement of hypesthesia in the left LE region	

AROM = active range of motion, EQ-5D = EuroQol 5 Dimension, LE = lower extremity, Lt = left, NRS = numerical rating scale, ODI = Oswestry Disability Index, PROM = passive range of motion, Rt = right, SLRT = Straight Leg Raise Test.

At the patient’s first admission on December 23, 2021, the NRS score for radicular pain in both LEs was 10, and the Oswestry Disability Index (ODI) score was 80. The patient also had hypesthesia (numbness) in the leg, which led to him to feel only 40% of his normal sensation. The result of the Straight Leg Raise Test (SLRT) was 20/20. At the second admission to the hospital postoperatively, on February 10, 2022, radicular pain in both LEs had decreased, and the patient had an NRS score of 5 and an ODI score of 30, showing overall improvement. A 40/40 SLRT result was obtained. Outpatient treatment was initiated on February 19, 2022, and overall improvement was noted, with the NRS score decreasing to 4 and ODI score to 11. However, there was a significant decline in the SLRT active range of motion at 20/20 and passive range of motion at 30/30. Additionally, a severe tingling sensation was present on the soles of both feet, and hypesthesia (numbness) behind both thighs was aggravated.

On June 25 2022, the NRS score improved to 2; the range of motion of the patient increased to within the normal range, with an active range of motion of 50/50 and a passive range of motion of 60/60 in the SLRT results. The strength of the LE also increased.

On September 3, 2022, the patient received combined treatment using Korean and conventional medicine, achieving improvement in the overall symptoms. Thereafter, the patient’s symptoms were managed with only physical and manual therapy. On August 1, 2023, the NRS score decreased to 1, and hypesthesia was slightly improved. Subsequently, the patient received only manual therapy for ongoing symptomatic management.

## 4. Discussion

### 4.1. Literature review

#### 4.1.1. IKM treatment for managing complications following LDH surgery

FBSS is a common complication following lumbosacral spine surgery, characterized by severe, chronic, and disabling LBP, with or without radicular pain, which remains resistant to physical therapy and pharmacological treatment.^[25]^ With an increasing proportion of patients undergoing surgery for LBP,^[26,27]^ the incidence of FBSS, in which patients complain of persistent postoperative pain or discomfort, varies widely (10–40%).^[28]^ Patients with FBSS often experience a more significant decline in quality of life than those with other conditions,^[29]^ making FBSS a significant social concern.

Considering the multifactorial nature of the etiology of FBSS, multidisciplinary approaches have been recommended for management.^[30]^ IKM treatment may be effective for patients with FBSS. According to a retrospective study with a 16-week IKM treatment program for FBSS, 89.4% of patients reported improvement in symptom outcomes at the 6-month follow-up, and 79.2% showed improvement at the 1-year follow-up, with reduced LBP and improved quality of life.^[14]^ Furthermore, the results of the long-term follow-up of patients with FBSS showed a reduction in the ODI score over time.^[15]^

However, the efficacy of IKM treatment for FBSS remains debatable due to the lack of high-quality evidence. A meta-analysis on the effectiveness of electroacupuncture in treating FBSS reported that electroacupuncture had no significant effect on pain reduction, and the quality of evidence in related randomized controlled clinical trials (RCTs) was low.^[31]^ A study showed that IKM treatment outcomes for patients who developed FBSS after lumbar spinal surgery were not significantly better in terms of the EuroQol 5 Dimension, ODI, and NRS scores than those of the control group.^[32]^ Therefore, there is a pressing need to accumulate high-quality evidence on the long-term effects of IKM treatment on FBSS.

#### 4.1.2. Vaccine side-effects: musculoskeletal inflammation/immune-mediated effects

The timeframe considered to be likely related to an infection or vaccination is measured from the onset of the event up to 6 weeks following the infection or vaccination.^[33]^ A postvaccination inflammatory response is the most common and anticipated AE. Among the types of inflammatory responses, local reactions at the injection site in the upper arm are the most frequent; however, systemic inflammatory responses can also occur. Excessive immune responses may lead to SAEs. The following are reports of SAEs that have occurred in the musculoskeletal system after COVID-19 vaccination to date.

#### 4.1.3. Intervertebral disc disease

RCTs for the evaluation of the safety and efficacy of the AZD1222 vaccine were conducted on 23,848 participants aged ≥ 18 years in the United Kingdom, Brazil, and South Africa from April 23 to November 4, 2020; the results of the safety follow-up reported 4 cases of LDH, categorized as severe musculoskeletal and connective tissue disorders.^[24]^ In an RCT conducted at 99 centers across the US on 30,420 volunteers, 2 cases of lumbar spinal stenosis were reported up to 28 days following messenger RNA (mRNA)-1273 vaccination.^[34]^

#### 4.1.4. Spondylitis

In an RCT for the evaluation of the safety and efficacy of the AZD1222 vaccine, one case of ankylosing spondylitis was reported, categorized as a severe musculoskeletal and connective tissue disorder.^[24]^ In a case series comprising reports from 16 different rheumatology centers in Italy between January and August 31, 2021, one case of spondylitis with sacroiliitis was reported.^[35]^ Additionally, a 17-year-old female patient was diagnosed with seronegative spondyloarthritis following the first dose of BNT162b2 vaccination.^[36]^

#### 4.1.5. Arthritis

In the report from 16 rheumatology centers in Italy, 66 individual cases of arthritis were described. Most patients (59%) received the BNT162b2 vaccine, and arthritis symptoms initiated at 11 to 13 days after the vaccination date. The most common clinical manifestations were girdle pain and stiffness resembling polymyalgia rheumatica (PMR), followed by oligoarthritis and polyarthritis.^[34]^ Another study reported 16 cases of arthritis related to COVID-19 vaccination.^[37]^

#### 4.1.6. Sacroiliitis

In an Italian study, a single case of sacroiliitis that developed after vaccination was reported.^[34]^ In another study, 3 days after administration of the Comirnaty vaccine, a pelvic scan of a female patient revealed inflammatory joint edges, bone erosion, and a heterogeneous mass, which was reported as a possible case of reactive unilateral sacroiliitis.^[38]^

#### 4.1.7. Rheumatoid arthritis

A 74-year-old woman with no history of arthritis or other rheumatic disease presented with complaints of swelling and pain in the right wrist, as well as the second to fourth metacarpophalangeal and proximal interphalangeal joints, which had been ongoing for 20 days. It had started 2 days after the patient had received the first dose of the Sinovac vaccine, and she was diagnosed with arthritis.^[39]^ In another study, a 53-year-old man with no history of rheumatoid arthritis was diagnosed with the disease 4 weeks after receiving the second dose of BNT162b2 vaccination.^[40]^

#### 4.1.8. Septic arthritis

A 45-year-old Thai woman was diagnosed with septic arthritis after experiencing left shoulder pain, limited range of motion, and fever following administration of the AstraZeneca/Oxford COVID-19 vaccine. Joint fluid culture showed *Staphylococcus aureus*.^[41]^ Additionally, another study reported a case of a 69-year-old African American man who developed calcium pyrophosphate deposition, flare, and septic arthritis 3 days after receiving a COVID-19 vaccination.^[42]^

#### 4.1.9. Rhabdomyolysis

An 80-year-old man experienced generalized body aches, nausea, and vomiting 2 days after receiving the second dose of the Moderna COVID-19 vaccine. He also had severe weakness and myalgia, and was diagnosed with rhabdomyolysis.^[43]^ Another study reported a case of a 58-year-old woman who developed severe rhabdomyolysis with weakness of the proximal and distal muscles, difficulty in walking, and reduced urine output after the third dose of the BNT162b2 vaccine.^[44]^ There have been other reports of post-COVID-19 vaccination-related rhabdomyolysis.^[45–48]^

#### 4.1.10. Polymyalgia rheumatica

A 69-year-old woman complained of sudden onset of bilateral pain in the shoulder and pelvic girdles with morning stiffness lasting more than 2 hours, fever, and general malaise the day after the first dose of COVID-19 vaccination and was diagnosed with PMR on the basis of her laboratory test results.^[49]^ Other reports have described cases where patients were diagnosed with PMR on the basis of the examination of bilateral pain in the shoulder and pelvic girdles, along with persistent fever following COVID-19 vaccination.^[50,51]^

#### 4.1.11. Benign fasciculation syndrome

In a previous report, a 48-year-old woman developed fasciculations that initially appeared as local symptoms 6 days after vaccination but later became generalized. She was diagnosed with benign fasciculation syndrome.^[52]^

#### 4.1.12. Parsonage–Turner syndrome

In a retrospective study, medical records of patients (mean age: 51 years) diagnosed with Parsonage–Turner syndrome (PTS) following COVID-19 vaccination in 3 hospitals in Korea between June and October 2021 were reviewed. In all cases, vaccination was the only identified cause for the onset of PTS. Apart from 2 patients, all developed symptoms after the first dose of the vaccine.^[53]^ One report detailed the case of a 40-year-old woman who was diagnosed with PTS 1 month after receiving the second dose of the BNT162b2 vaccine.^[54]^ Additionally, there was a report of 2 patients who developed PTS 13 hours and 18 days after BNT162b2 vaccination.^[55]^

#### 4.1.13. Shoulder injury related to vaccine administration

Shoulder injury related to vaccine administration is a post-COVID-19 vaccination complication with symptoms of shoulder pain and limited range of motion after injection of the vaccine into the subdeltoid bursa^[56,57]^; an 83-year-old woman was diagnosed with Shoulder injury related to vaccine administration following BNT162b2 vaccination.^[58]^

Severe immune-mediated AEs involving nervous system disorders may also occur after COVID-19 vaccination. Central or peripheral nervous system disorders may also result in musculoskeletal disorders.

#### 4.1.14. Guillain–Barre syndrome

In 2021, 815 reports of Guillain–Barre syndrome (GBS) and its variants were filed as AEs following COVID-19 vaccination in the VAERS of the US. The mean age of the patients was 55 years, and 50% were men. The median time to onset was 10 days, and 77% of the patients received inpatient care, of which lack of recovery, permanent disability, and death constituted 57%, 46%, and 2% of outcomes, respectively.^[59]^ A 46-year-old Iranian man developed ascending weakness with pain in his lower limbs 3 days after receiving the second dose of the AstraZeneca/Oxford vaccine; his deep tendon reflexes were absent, and he was diagnosed with GBS. Other studies have also reported cases of postvaccination GBS.^[60–65]^

#### 4.1.15. Myasthenia gravis

A 33-year-old woman presented to the emergency department with bilateral ptosis and binocular diplopia following a second dose of the BNT162b2 vaccine. The patient was diagnosed with myasthenia gravis based on the results of a neostigmine test and electromyography.^[66]^ A case series also reported the cases of 3 patients with new-onset myasthenia gravis presenting with ocular and bulbar symptoms following COVID-19 vaccination.^[67]^

#### 4.1.16. Acute transverse myelitis

A case report in Korea presented 2 cases as follows. In the first case, an 81-year-old man received the BNT162b2 vaccine on April 29 and May 19, 2021; at 3 days after receiving the second dose, the patient presented with bilateral hand weakness and was diagnosed with acute transverse myelitis (ATM) at the C1 to C3 level based on MRI findings. In the second case, a 23-year-old woman received the first dose of the BNT162b2 vaccine on August 18, 2021. At 3 weeks after the vaccination, she had sudden onset of tingling in both thighs, followed by weakness in legs after 1 hour; she was diagnosed with ATM.^[68]^ In early March 2021, a 44-year-old woman who had received the first dose of the AZD1222 vaccine developed minor symptoms; 4 days thereafter, she complained of ascending paresthesia, noticed reduced sensation during micturition, and was diagnosed with ATM.^[69]^ Multiple other cases of ATM following COVID-19 vaccination have been reported.^[70–72]^

#### 4.1.17. Functional neurological disorders

At a rehabilitation center in Spain, 6 individuals who developed sequelae after COVID-19 vaccination were diagnosed with functional neurological disorders, presenting with various symptoms. Most patients experienced the onset of symptoms within 4 weeks of COVID-19 vaccination. Among these patients, one developed bilateral leg weakness and shaking, which impaired gait and necessitated the use of a wheelchair for mobility.^[73]^

#### 4.1.18. Immune-mediated demyelination

A 38-year-old man presented with sensory loss and bladder disturbance, along with weakness in both lower limbs, the day after receiving the first dose of the AZD1222 vaccine. Additionally, a 50-year-old man experienced difficulty in walking for 11.5 weeks after the administration of COVAXIN. Another 38-year-old man presented with subacute onset of progressive symmetric quadriparesis 2 months after receiving the first dose of his COVID-19 vaccine. These were all reported as cases of postvaccination immune-mediated demyelination.^[74]^

### 4.2. Vaccine side effects: aggravation or recurrence of preexisting conditions

Cases of recurrence or aggravation of preexisting conditions and/or underlying diseases induced by systemic inflammatory responses to COVID-19 vaccination have also been reported.

An 18-year-old man had been diagnosed with systemic onset juvenile idiopathic arthritis (Still disease) 3 years prior but had undergone treatment to achieve remission; however, after receiving 2 doses of the AZD1222 vaccine on August 28 and November 29, 2021, he developed a sore throat, dyspnea, myalgia, and high-grade fever, and his clinical examination revealed high total blood counts, high erythrocyte sedimentation rates, increased C-reactive protein levels, and elevated ferritin levels; thus, he was diagnosed with a flare-up of Still disease.^[75]^ An 82-year-old woman in the Netherlands had been diagnosed with hepatitis C infection in 2007 but had recovered fully in 2011. On day 3 (February 21, 2021) after administration of her first dose of the BNT162b2 vaccine, she developed jaundice; on day 23, she lost consciousness and died, and the results of a postmortem blood test confirmed hepatitis C.^[76]^ Moreover, a 54-year-old woman, who had been diagnosed with immunoglobulin A nephropathy 16 years prior, developed gross hematuria 2 days after her second dose of the mRNA-1273 vaccine; kidney biopsy showed immunoglobulin A staining, and she was diagnosed with a relapse of immunoglobulin A nephropathy.^[77]^

A case report examined the temporal association between COVID-19 vaccination and relapses of multiple sclerosis (MS). Sixteen patients with MS who received COVID-19 vaccination presented with confirmed relapses between March and June 2021; those with a history of MS developed MS relapses 3 days to 3 weeks after the first dose of vaccination, suggesting a temporal association between MS activity and COVID-19 vaccination.^[78]^ A 36-year-old woman in China who had been diagnosed with left-sided Bell palsy 18 years prior was diagnosed with recurrent right-sided Bell palsy 2 days after her first dose of the Sinovac vaccine.^[79]^ Of the 732 patients diagnosed with GBS in Israel between 2000 and 2020, 48 experienced recurrence following BNT162b2 vaccination.^[80]^

### 4.3. Interpretation of the case findings

In this patient, recurrent LDH occurred after COVID-19 vaccination. Although few case reports of rDH after COVID-19 exist, there are many reports of different types of musculoskeletal inflammation or immune-mediated side effects following vaccination. Recurrence of preexisting conditions due to overactivation of immune responses has also been reported. In our case, the patient had a medical history of LDH. This patient developed rDH symptoms 2 days after COVID-19 vaccination, and no other excessive activity that could be suspected of causing rDH was performed after vaccination. The patient attributed the symptoms to the vaccine, noting that they began to recur on Sunday, after a weekend of sufficient rest, following vaccination on Friday. Considering the patient’s circumstances and previous reports on AEs following vaccination, we cannot rule out the possibility of COVID-19 vaccination causing rDH.

The mechanisms of vaccination-induced AEs vary depending on the type of COVID-19 vaccine. Here, the BNT162b2 vaccine administered to the patient used nucleoside-modified mRNA to produce proteins that induced robust immune responses.^[81]^ In this process, lipid nanoparticles are broadly distributed in human tissues and organs, and AEs may be related to the proinflammatory action of lipid nanoparticles or the delivered mRNA in areas of preexisting comorbidities.^[82]^ The risk of AEs is also associated with increasing pro-inflammatory cytokine/inflammatory chemokine signatures with the upregulation of tumor necrosis factor-α and interleukin-6 following the BNT162b2 vaccination.^[83]^ Upon vaccination, the virus manipulates the host cellular machinery and ribosomes to replicate the severe acute respiratory syndrome coronavirus 2 spike protein and trigger immune responses. During this process, the mRNA vaccine identifies the spike protein as a foreign antigen, triggering a proinflammatory cytokine cascade. This mechanism is similar to that of neurotoxicity, leading to elevated cytokine levels and secondary damage to organs.^[84]^ Considering the findings of previous studies, in this patient, recurrence and aggravation of the LDH may have occurred owing to increased inflammatory responses in the vulnerable preexisting LDH area that were activated by the BNT162b2 vaccination.

IKM treatment, comprising acupuncture, herbal medicine, pharmacopuncture, and CMT, was administered to a patient with FBSS who underwent surgery for severe recurrent LDH, resulting in improved outcomes. Cheongpajeon, the main herbal medicine used, contains medicinal herbs. GCSB-5, the primary ingredient of Cheongpajeon, is effective in reducing acute and chronic inflammation; its anti-inflammatory activity is likely attributed to the inhibition of inducible nitric oxide synthase and cyclooxygenase-2 expression.^[85]^ Pharmacopuncture involved the use of Shinbaro pharmacopuncture solution, the main ingredient of which is also GCSB-5. CMT is a manual therapy in Korean medicine in which the specialist applies effective stimulation to the affected area of the body of a patient using their hands or other tools.^[86]^ It is a safe and effective Korean medicine treatment for reducing pain and improving functional outcomes in patients with musculoskeletal disorders.^[86,87]^

As this study reports on a single case, the results alone are insufficient to clearly establish a causal relationship between COVID-19 vaccination and recurrent LDH or the effectiveness of IKM treatment on postoperative symptoms following LDH surgery. Nevertheless, it seems possible that vaccination and LDH recurrence are related because they are temporally related and existing studies support that there is a possibility of LDH and preexisting disease intensification after vaccination. In addition, this study provides evidence on the long-term effects of IKM treatment for the sequelae of CES surgery, offering new insights into its potential benefits for managing this condition.

In the post-COVID-19 era, as the outbreak shifts from the pandemic to an endemic state, postvaccination SAEs should be carefully evaluated to help maintain vaccine efficacy while minimizing social costs. Further in-depth analyses and a thorough understanding of the side effects of COVID-19 vaccines are required before incorporating COVID-19 vaccination into any national immunization program.

## 5. Conclusions

This paper confirmed the possibility that COVID vaccination is related to LDH recurrence and the possibility of IKM as an effective treatment option after LDH surgery. Careful consideration and consultation are recommended before COVID-19 vaccination.

## Author contributions

**Conceptualization:** Ah-Ra Koh, Doori Kim.

**Investigation:** Ah-Ra Koh, Hyun-Woo Kim, Young-Jin Lee, Hye-Jeong Jo, Go-Eun Chae, Dong-Woo Kim.

**Methodology:** Doori Kim.

**Project administration:** In-Hyuk Ha.

**Supervision:** In-Hyuk Ha.

**Writing – original draft:** Ah-Ra Koh.

**Writing – review & editing:** Ah-Ra Koh, Doori Kim.

## Supplementary Material


