# Supplementary material for: Integrative Korean medicine for recurrent lumbar disc herniation after coronavirus disease vaccination: A case report and literature review
Source: Medicine (Baltimore). 2025 Jan 3;104(1):e41079. doi: 10.1097/MD.0000000000041079 (PMC11709196; doi:10.1097/MD.0000000000041079)
Supplement: Supplementary file 1 [file medi-104-e41079-s001.docx]

**Supplemental Digital Content 1.** **Ingredients of intervention during 1^st^ Inpatient Treatment**

| Treatment type | Ingredients |
| --- | --- |
| Herbal medicine | |
| Cheongpajeon(CPJ) | *Eucommia ulmoides* 7.5 g, *Acanthopanax sessiliflorus* 7.5 g, *Achyranthes bidentata* 7.5 g, *Saposhnikovia divaricata* 7.5 g, *Cibotium barometz* 7.5 g, *Lycium chinense* 7.5 g, *Boschniakia rossica* 7.5 g, *Cuscuta chinensis* 7.5 g, *Glycine max* 7.5 g, *Ostericum koreanum* 3.75 g, *Atractylodes japonica*. 3.75 g, *Psoralea corylifolia* 3.75 g |
| Chungsinbaro-hwan | *Poria cocos (Schw.) Wolf* 0.156g, *Ginseng Radix Alba* 0.078g*, Achyranthes bidentata Bl.* 0.052g, *Asini Gelatinum* 0.019g*, Rehmanniae Radix* 0.623g, *Cervi Cornus Colla* 0.065g*, Mel* 0.312g, *Cibotii Rhizoma* 0.052g, *Eucommiae Cortex* 0.026g*, Saposhnikovia Radix* 0.013g*, Acanthopanacis Cortex* 0.013g, *Scolopendra Corpus* 0.013g, *Atractylodis Rhizoma Alba* 0.052g, *Bovis Fel.* 0.026g |
| Gwanjul-go | *Poria cocos (Schw.) Wolf* 1.634g, *Ginseng Radix Alba* 0.817g*, Achyranthes bidentata Bl.* 0.204g, *Asini Gelatinum* 0.102g*, Rehmanniae Radix* 3.268g, *Cervi Cornus Colla*  0.34g*, Mel* 1.634g |
| Madae-hwan | *Rhei Radix et Rhizoma* 4.0g, *Ponciri Fructus Immaturus* 2.0g*, Armeniacae Semen* 2.0g*, Magnoliae Cortex* 2.0g, *Paeoniae Radix* 2.0g*, Cannabis Semen* 5.0g |
| Pharmacopuncture | |
| Shinbaro (Jaseng Korean Medical Hospital, Namyangju, Republic of Korea) | Cibotium barometz, Saposhnikovia divaricata , Eucommia ulmoides, Acanthopanax sessiliflorus, Ostericum koreanum, Angelica pubescens, Achyranthes japonica, Paeonia albiflora, Scolopendra subspinipes |

**Supplemental Digital Content 2. Details of intervention during 2^nd^ Inpatient Treatment.**

| Treatment type | Ingredients |  |
| --- | --- | --- |
| Herbal medicine | | |
| Cheongpajeon (CPJ) | *Eucommia ulmoides* 7.5 g, *Acanthopanax sessiliflorus* 7.5 g, *Achyranthes bidentata* 7.5 g, *Saposhnikovia divaricata*, 7.5 g, *Cibotium barometz* 7.5 g*, Lycium chinense* 7.5 g, *Boschniakia rossica* 7.5 g, *Cuscuta chinensis* 7.5 g, *Glycine max* 7.5 g, *Ostericum koreanum* 3.75 g, *Atractylodes japonica.* 3.75 g, *Psoralea corylifolia* 3.75 g | |
| Pharmacopuncture | | |
| Shinbaro (Jaseng Korean Medical Hospital, Namyangju, Republic of Korea) | *Cibotium barometz, Saposhnikovia divaricata, Eucommia ulmoides,*  *Acanthopanax sessiliflorus, Ostericum koreanum, Angelica pubescens,*  *Achyranthes japonica, Paeonia albiflora, Scolopendra subspinipes* | |

**Supplemental Digital Content 3. Details of intervention during Outpatient Treatment.**

| Treatment type | Ingredients |
| --- | --- |
| Herbal medicine | |
| Cheongpajeon (CPJ) | *Eucommia ulmoides* 7.5 g, *Acanthopanax sessiliflorus* 7.5 g, *Achyranthes bidentata* 7.5 g, *Saposhnikovia divaricata*, 7.5 g, *Cibotium barometz* 7.5 g*, Lycium chinense* 7.5 g, *Boschniakia rossica* 7.5 g, *Cuscuta chinensis* 7.5 g, *Glycine max* 7.5 g, *Ostericum koreanum* 3.75 g, *Atractylodes japonica.* 3.75 g, *Psoralea corylifolia* 3.75 g |
| Pharmacopuncture | |
| Shinbaro (Jaseng Korean Medical Hospital, Namyangju, Republic of Korea) | *Cibotium barometz, Saposhnikovia divaricata, Eucommia ulmoides,*  *Acanthopanax sessiliflorus, Ostericum koreanum, Angelica pubescens,*  *Achyranthes japonica, Paeonia albiflora, Scolopendra subspinipes* |
